# Zonal control on Holocene precipitation in northwestern Madagascar based on a stalagmite from Anjohibe

**DOI:** 10.1038/s41598-024-55909-6

**Published:** 2024-03-06

**Authors:** Robin R. Dawson, Stephen J. Burns, Benjamin H. Tiger, David McGee, Peterson Faina, Nick Scroxton, Laurie R. Godfrey, Lovasoa Ranivoharimanana

**Affiliations:** 1 Department of Earth, Geographic and Climate Sciences, University of Massachusetts Amherst, Amherst, MA 01003 USA; 2 Department of Earth, Atmospheric, and Planetary Sciences, Massachusetts Institute of Technology, Cambridge, MA 02139 USA; 3 Department of Geology and Geophysics, Woods Hole Oceanographic Institution, Woods Hole, MA 02543 USA; 4 The Climate School, Columbia University, New York, NY 10025 USA; 5 Irish Climate Analysis and Research Units, Department of Geography, Maynooth University, Maynooth, Ireland; 6 Department of Anthropology, University of Massachusetts, Amherst, MA 01003 USA; 7 Mention Bassins Sédimentaires, Evolution, Conservation, Faculté des Sciences, Université D’Antananarivo, Antananarivo, Madagascar

**Keywords:** Palaeoclimate, Hydrology

## Abstract

The Malagasy Summer Monsoon is an important part of the larger Indian Ocean and tropical monsoon region. As the effects of global warming play out, changes to precipitation in Madagascar will have important ramifications for the Malagasy people. To help understand how precipitation responds to climate changes we present a long-term Holocene speleothem record from Anjohibe, part of the Andranoboka cave system in northwestern Madagascar. To date, it is the most complete Holocene record from this region and sheds light on the nature of millennial and centennial precipitation changes in this region. We find that over the Holocene, precipitation in northwestern Madagascar is actually in phase with the Northern Hemisphere Asian monsoon on multi-millennial scales, but that during some shorter centennial-scale events such as the 8.2 ka event, Anjohibe exhibits an antiphase precipitation signal to the Northern Hemisphere. The ultimate driver of precipitation changes across the Holocene does not appear to be the meridional migration of the monsoon. Instead, zonal sea surface temperature gradients in the Indian Ocean seem to play a primary role in precipitation changes in northwestern Madagascar.

## Introduction

**Figure 1:**
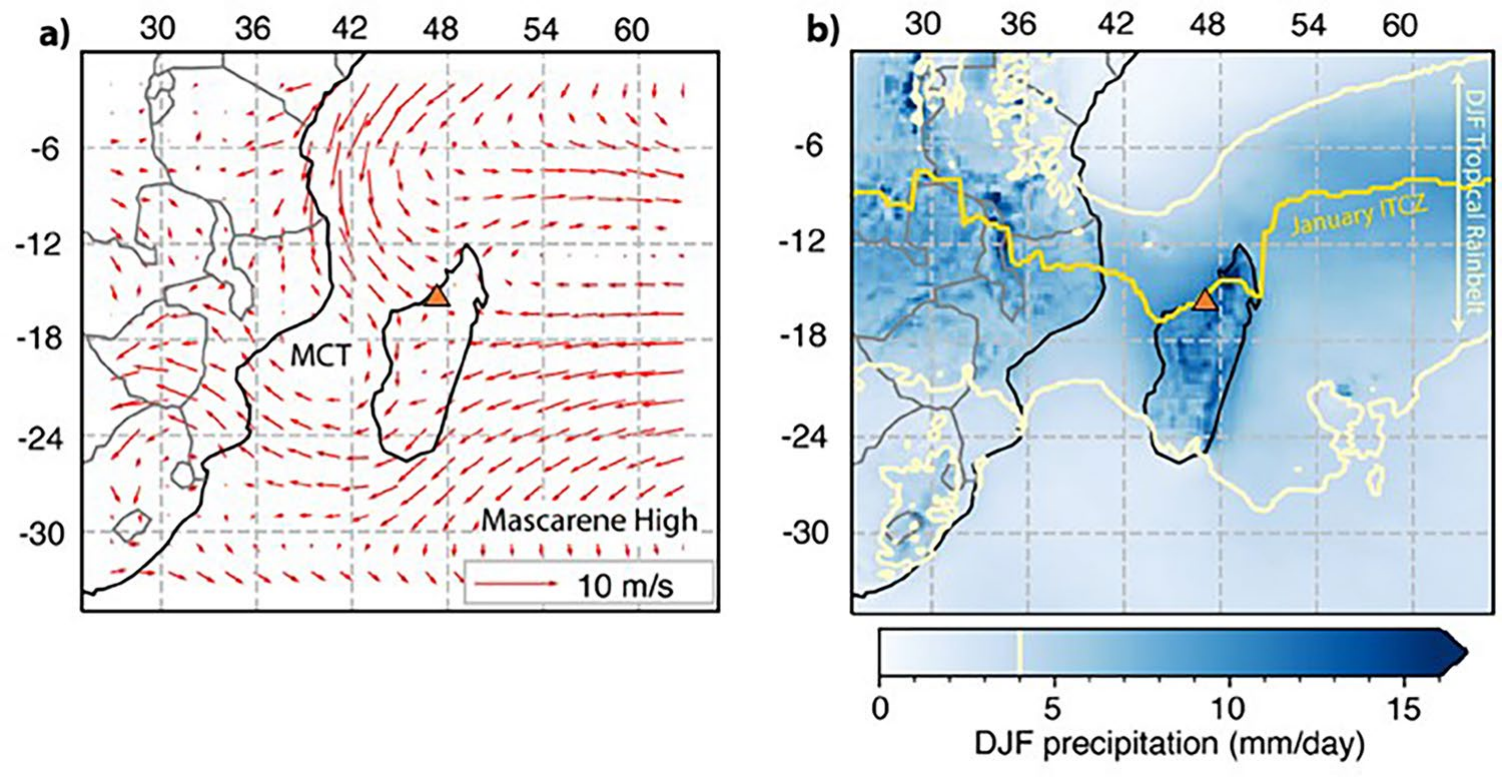
Wind and precipitation for stalagmite collection site, Anjohibe (orange triangle). (**a**) ERA5 (fifth generation European reanalysis data) at the 850 hectopascal (hPa) atmospheric pressure level with January mean wind direction and strength (red arrows)^85^ with Mozambique Channel Trough (MCT) and Mascarene High labeled. (**b**) ERA5 DJF mean rainfall (blue gradient) with 4 mm isohyet (light yellow line) to denote approximate extent of the tropical rain belt and ERA5 outgoing longwave radiation minima (bright yellow line) to denote approximate position of the summer ITCZ^86^. ERA5 data^85–87^ was accessed through the Copernicus Climate Data Store (https://cds.climate.copernicus.eu/) and maps generated with Python version 3 and Cartopy package version 0.20.0.

In future decades tropical countries will bear the brunt of the impacts of anthropogenic climate change. Madagascar is particularly vulnerable, as 70% of the population are subsistence farmers, and food insecurity is projected to worsen with increased frequency of extreme weather events affecting crops and livestock^1^. As the world’s 4th largest island with 80–100% endemic plant and animal species^2^, climate hazards including cyclones, floods, and droughts put the nation’s biodiversity at risk as well as human health and infrastructure^3,4^. Thus, to help develop climate-resilient policies, it’s important to understand what drives precipitation changes. In today’s climate, a rain shadow is created by the mountains that run along the eastern margin of Madagascar. Air masses transported by southeasterly trade winds drop their moisture along these eastern highlands^5^. Therefore, the main source of moisture to Anjohibe is the seasonal monsoon rains delivered by northwesterly winds retroflected from highlands in eastern Africa during the austral summer, with minor amounts of moisture coming from the southeasterly trade winds during the dry season^6^ (Fig. 1). These monsoon rains, controlled by seasonal insolation changes and the migration of the Inter-Tropical Convergence Zone (ITCZ) and tropical rain belt, occur from December through February and make up 70% of the total annual rainfall^5–7^. The latitudinal extent of the tropical rain belt also produces a general north–south precipitation gradient (Fig. 1) with as much as 450 mm of rain falling in the summer months in the north and at most 200 mm in the south^7^.

Inter-annual variability of precipitation is less well understood as climate station data coverage from year to year is inconsistent. The Indian Ocean Dipole (IOD), a change in the west to east sea surface temperature (SST) gradient and associated changes to surface winds, similar to the El Niño-Southern Oscillation (ENSO), affects precipitation in the region today^8,9^. During a positive IOD event, increased upwelling off Sumatra produces anomalously cool SSTs in the eastern Indian Ocean, leading to strengthened easterlies, a weaker zonal SST gradient and more convective rainfall over the western Indian Ocean near Madagascar^8,10^. The IOD has been coupled to ENSO variability over the last millennium^10^ and though not always the case, a strong El Niño might prime the Indian Ocean for a positive IOD event^9^. During the negative phase of the IOD, colder than average SSTs in the western Indian Ocean strengthen the zonal SST gradient producing wetter conditions in Australia and Indonesia^10^. Today, negative IODs have a smaller amplitude than positive events likely due to the deep thermocline in the eastern Indian Ocean^11^.

Annual layer thickness data from Anjohibe (“Big cave” in Malagasy) going back to 1550 AD suggest warmer Indian Ocean SSTs and higher ENSO frequency are associated with increased precipitation in northwestern Madagascar on centennial timescales^12^. On multi-decadal to multi-centennial timescales, proxy data and model outputs for the last millennium show that Indian Ocean SSTs were the primary control on East African precipitation^13^, with an IOD-like inverse relationship between precipitation in East Africa and Indonesia^14^. However, the relationship between the IOD and precipitation has been shown to be non-stationary on different timescales, likely due to teleconnections with the Asian monsoon, Pacific Ocean and Indo-Pacific Warm Pool (IPWP) SSTs^14,15^.

On multi-millennial timescales, it is thought that the Northern and Southern Hemisphere tropics exhibit antiphase climate signals due to precession-paced changes in insolation and the consequent migration of the mean position of the ITCZ north or south of the equator in a “Global Paleomonsoon”^16,17^. Over centennial timescales, a record from the last 1700 years from Anjohibe (Fig. 1) suggests that precipitation variations north and south of the equator in the tropical western Indian Ocean were in phase^6^. This record and others from the late Holocene Indo-Pacific region that also show in phase precipitation variations suggest that the tropical rain belt was expanding and contracting during these times^18,19^. However other records from Australia (KNI-51) and China (Dongge Cave) display an antiphase relationship in monsoon rainfall across the two hemispheres^20^. This varying response of the tropical rain belt is likely due to regional differences in surface type (land vs ocean) and continental configuration which override the inter-hemispheric temperature gradient forced meridional shift^21^.

Our understanding of past precipitation changes in northern Madagascar is based largely on speleothem stable isotope (δ^18^O and δ^13^C) records from Anjohibe (− 15.542° latitude, 46.885° longitude), a cave located about 70 km northeast of Mahajanga and formed in the Eocene Narinda Limestone^22,23^. Most speleothem records only reflect deposition within the middle- to late Holocene^6,24–29^, but one longer Holocene record exists, with several hiatuses^30^. Further north, a longer term record covering the Holocene and last Glacial Termination is based on lake sediments and pollen from Lac Maudit, although compared to Anjohibe, this is a high elevation site (1250 m.a.s.l.)^31^. The speleothem and lake records show a wetter early Holocene and a drier late Holocene, which is contrary to what is expected, assuming that precipitation is controlled by local summer insolation.

To help answer remaining questions about drivers of paleoclimate in northwestern Madagascar, we present the results of a nearly complete Holocene record of climate from Anjohibe based on stalagmite AB11 collected in October 2019. The δ^18^O values of stalagmite calcium carbonate reflect the temperature-dependent fractionation between water and calcium carbonate and the δ^18^O composition of the formational drip waters^32^. The δ^13^C values of stalagmites are controlled by the isotopic signatures of dissolved inorganic carbon (DIC) and gaseous CO_2,_ the stalagmite’s growth rate, and the supersaturation state of the formation waters with respect to calcium carbonate^33^. In a well-ventilated cave within a dry climate like Anjohibe, climate controls on stalagmite δ^13^C values reflect the three carbon sources: atmospheric CO_2_, soil CO_2_, and dissolution of the karst bedrock^32,33^. Therefore, a record of stalagmite stable isotopes (δ^13^C and δ^18^O) should reflect climatic information from the time period it formed.

## Results

### U/Th age model for AB11

**Figure 2:**
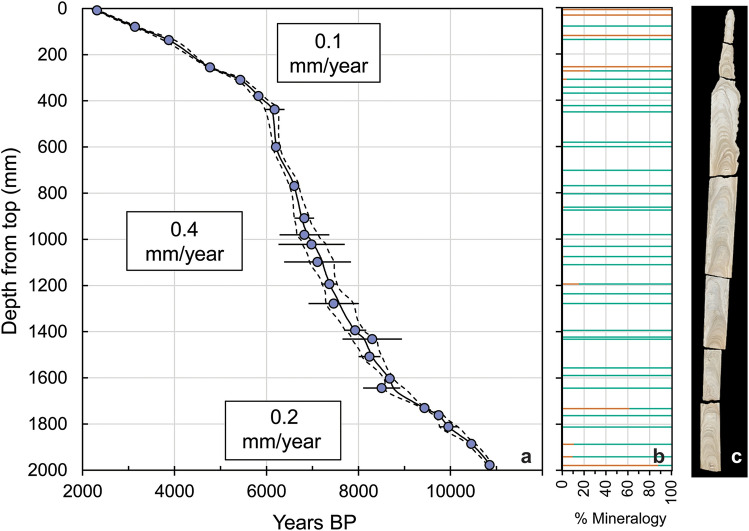
(**a**) Age-depth relationship for speleothem AB11. 1000 Monte-Carlo simulations were run in COPRA to generate median ages and confidence intervals^84^. The median age is shown as a solid black line, and the 95% confidence interval is shown with dashed lines. U-Th ages used for the model are shown in purple. Error bars represent the 2σ uncertainties for each age determination. (**b**) Selected depths sampled for mineralogical analysis using XRD. % Aragonite in orange and % calcite in blue green. (**c**) Photograph of AB11 approximating depths of samples analyzed in (**a**) and (**b**).

Stalagmite AB11 grew continuously from 10.9 ky BP to 2.3 ky BP based on 25 U/Th ages (Fig. 2A). The growth rates show two pronounced changes, with initial deposition of carbonate at 0.2 mm/year from 10.9 ky BP to 8.5 ky BP, a faster growth rate of 0.4 mm/year from 8.5 ky BP to 6 ky BP, and a return to slower growth of 0.1 mm/year from 6 ky BP to 2.3 ky BP. (Fig. 2). This latter inflection point coincides with the depth at which the stalagmite’s diameter decreases abruptly (Fig. 2c).

### Mineralogy of stalagmite AB11

Based on powder X-ray diffraction (XRD) of 38 samples, the mineralogy of AB11 is predominantly calcite. The bottom 30 cm show evidence of mixed aragonite calcite layers, while the upper 30 cm have more aragonite. The upper portion has entire layers of aragonite with alternating layers of calcite, which coincides with the change in diameter for the stalagmite and much slower growth rate (Fig. 2). Both sections with more aragonite coincide with the slower growth periods of AB11.

### Stable isotopes of AB11

**Figure 3:**
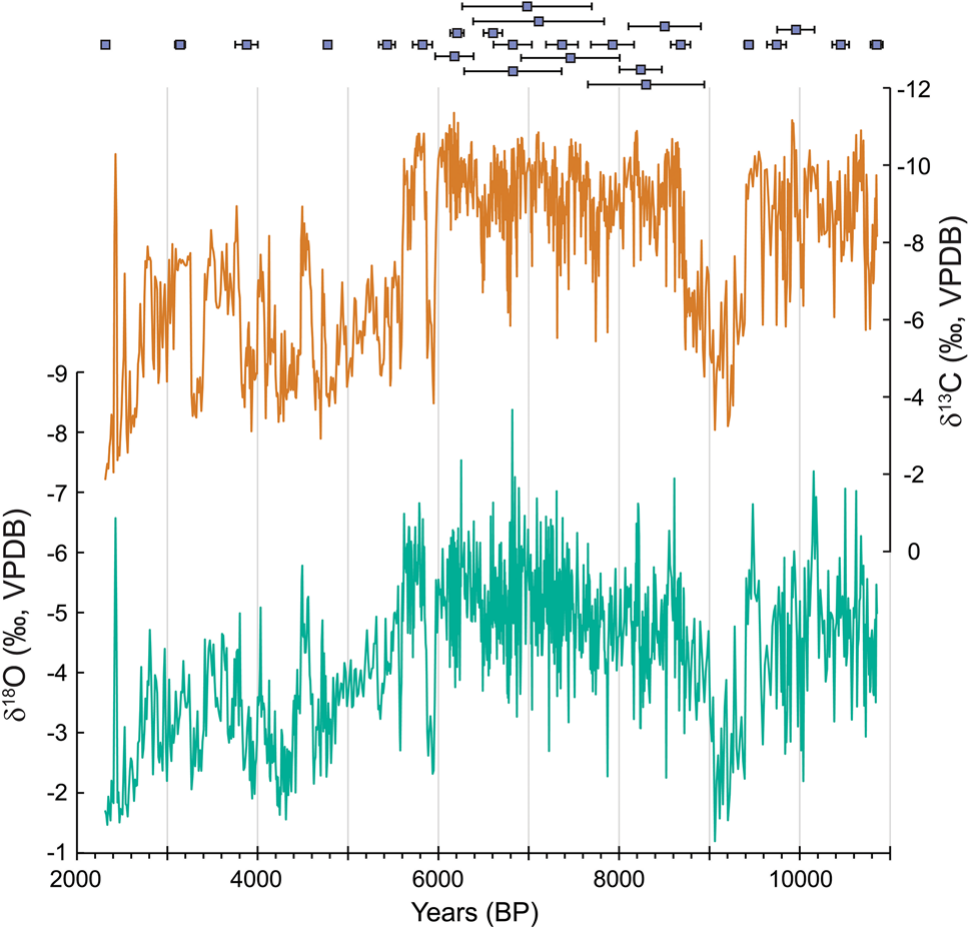
Stable carbon and oxygen isotope values of AB11. Top: U/Th ages (purple squares) with 2σ error bars, offset to show overlap. Middle: δ^13^C (‰, VPDB) values of AB11 in orange. Bottom: δ^18^O (‰, VPDB) values of AB11 in blue green.

A total of 1184 isotopic samples was taken every 2 mm from the stalagmite’s base at 1978 mm to 390 mm depth and then every 1 mm from 388 mm to the top. The samples were analyzed on an isotope ratio mass spectrometer (IRMS) for δ^18^O and δ^13^C and the values were plotted versus time in Fig. 3. Time resolution of the stable isotope samples for each of the three growth segments (from bottom to top) is about 11 years (1978–1646 mm), 5 years (1644–390 mm) and 9 years (388–1 mm) respectively. The δ^18^O values vary between − 8.4‰ and − 1.2‰ and the δ^13^C values between − 11.4‰ and − 1.9‰. Throughout the record δ^18^O and δ^13^C are positively correlated (ρ = 0.81, p ≪ 0.001). In general, lower values (average δ^18^O = − 4.9‰, average δ^13^C = − 9.0‰) are recorded in the older part of AB11 (~ 10.9 to 5.6 ky BPB), compared to higher values (average δ^18^O = − 3.1‰, average δ^13^C = − 5.6‰) in the younger part of the record (~ 4.6 ky BP to 2.3 ky BP). The transition from more to less negative values takes place over a time span of about 1000 years, from ~ 5.8 ky BP to 4.8 ky BP for δ^18^O. Several multi-decadal to multi-centennial excursions in isotopic values appear in the record. During the earlier part of the record, when more negative values are observed, the period from ~ 9.4 to 9.0 ky BP is marked by values enriched by about 3‰ in δ^18^O and 6‰ in δ^13^C (max δ^18^O = − 1.2‰, max δ^13^C = − 3.2‰). A second large positive excursion is found between 6.0 to 5.85 ky BP (max δ^18^O = − 2.3‰, max δ^13^C = − 3.8‰) with isotopic values in this interval similar to values in the younger portion of the record. After the transition from the more negative (older) part of the record to less negative (younger) part of the record, several negative isotopic excursions of at least 2‰ in δ^18^O and 4‰ in δ^13^C are found and lasting from several decades to about one century.

Wavelet analysis (see methods) of the δ^18^O values reveals significantly (at the 90% confidence level) more decadal to multi-decadal variability with ~ 15 to 120-year periodicity in the middle to older part of the record (≥ 5.6 ky BP; Supplementary Fig. S1). However, this could be partially due to lower isotope sampling resolution (9 years) in the youngest (1–388 mm, 2314–5877 years BP) part of the record compared to 5 years in the middle (390–1644 mm, 5888–8762 years BP).

## Discussion

### Paleoclimate interpretation of stable isotopes

In Madagascar and other tropical regions with monsoonal climates, the amount effect^34^ is interpreted to be the dominant control on δ^18^O values of meteoric precipitation^6,25,26,29,32,35–37^. Recent investigation of the physical mechanisms leading to the amount effect reveals a complex system with sub-cloud base microphysics and cloud type important in determining the isotopic composition of rainfall^38^. In spite of these complexities, measurements and modeling of isotopes in modern precipitation have found a strong inverse correlation between summer (December–February, DJF) δ^18^O and precipitation in northwest Madagascar and in the surrounding region^39^. Further, the amount effect is observed in interannual variations in DJF rainfall δ^18^O in central Madagascar (Antananarivo) and further east at Rodrigues and Mauritius islands based on the Global Network of Isotopes in Precipitation^27^ suggesting that this process is dominant across the tropical southwest Indian Ocean. Northwest Madagascar's summer rainfall is sourced almost exclusively from the equatorial western Indian Ocean^6^ and the study area is near the source. In addition, temperature has not varied much over the Holocene^40^, and a recent cave monitoring study at Anjohibe found that within cave temperatures are very stable, with fluctuations of ~ 2 °C or less within a season, equating to only ~ 0.4‰ change in δ^18^O values^41^ during a stalagmite growing period^42^. This same study also found that the δ^18^O values of Anjohibe drip waters reflect local mean rainfall δ^18^O values^42^ therefore the water composition largely controls the δ^18^O of the stalagmite^33^. We therefore interpret the Anjohibe δ^18^O record to reflect climate, namely precipitation and the intensity of the summer monsoon. Mineralogical changes from calcite to aragonite would change the AB11 δ^18^O values by − 0.8‰^43,44^. Estimating these mineralogical effects based on pure end-members and our XRD data (Supplementary Fig. S2) show that they are minimal compared to our isotopic shifts attributed to climate (~ 3‰). Given the large isotopic range and high correlation with δ^13^C, the magnitude of the signal in the speleothem carbonate is also likely exaggerated by kinetic fractionation effects.

In dry regions like Anjohibe, wetter conditions generally result in more vegetation, more plant respired versus atmospheric CO_2_ in the epikarst, more decay of organic matter, and minimal signal from the karst bedrock, all leading to more isotopically depleted δ^13^C values^32^. Consequently, δ^13^C values often have a negative relationship with precipitation, whereby wet and dry are reflected by more negative values and less negative values (respectively)^32,45^. Carbon isotope values can also reflect the difference in isotopic fractionation that occurs during the photosynthetic pathways of C_3_ plants such as woody taxa and C_4_ plants such as tropical grasses^46^. The former are generally favored in wetter conditions and are associated with more isotopically depleted values in secondary carbonates (− 14 to − 6‰); C_4_ grasses are reflected by more enriched secondary carbonate values (− 6 to + 2‰) and tend to grow under drier conditions^32,46^. For example, previous work in Anjohibe indicates a dramatic shift from C_3_ to C_4_ vegetation at about 1.1 ky BP, which is attributed to forest clearing by a growing human population practicing agropastoralism^29^. Changes in the C_3_/C_4_ ratio earlier in the Holocene are more likely to be climate-related. In addition to these primary climate drivers, δ^13^C values may be subject to kinetic fractionation effects related to fast carbonate growth rates and the extent of degassing^47^, which are more likely in well ventilated caves and lead to more enriched δ^13^C values^33^. Prior calcite (or aragonite) precipitation (PCP/PAP) can also affect the resulting speleothem δ^13^C values, where wetter conditions create faster flow rates through the karst system and less PCP (PAP), and thus more depleted δ^13^C values closer to the DIC of formation waters^45,47^. Similar to δ^18^O, pure aragonite layers would change δ^13^C values by − 1.7‰^48^ (Supplementary Fig. S2). In summary, carbon isotopes tend to broadly track oxygen isotopes as does our record from AB11, with cave processes often moving the δ^13^C signal in the same direction as other climate factors.

### Stable isotopes throughout the Holocene

The first order trend in the AB11 isotopic records shows a wetter early to middle Holocene, a transition to a drier climate beginning at about 6 ky BP and progressively drying climate toward the end of the record at 2.3 ky BP. This trend is opposite to that expected for the Southern Hemisphere (antiphase compared to Northern Hemisphere) based on the Global Paleomonsoon concept and to what has been observed in paleoclimate records of other monsoon regions^36,49–54^. Data and models suggest this antiphase behavior is driven by precessional changes in summer insolation, which shifts the mean position of the tropical rainfall belt and intensifies monsoons due to greater land-sea thermal contrasts^16,17^. Climate models show that when summer insolation is high in the Northern Hemisphere, the increased seasonality shifts the locus of convection (low near-surface atmospheric pressure) from near the equator northward. The opposite is true for Southern Hemisphere insolation maxima^55^. Regional differences have been demonstrated before, mostly due to ocean feedbacks, sometimes overriding the precipitation signal expected from insolation forcing alone such as in the Australian monsoon during the Holocene^56^. In addition, a 60,000-year lake record from tropical (~ 6°S) East Africa suggests that Northern Hemisphere insolation had an influence on precipitation in this region^57^.

**Figure 4:**
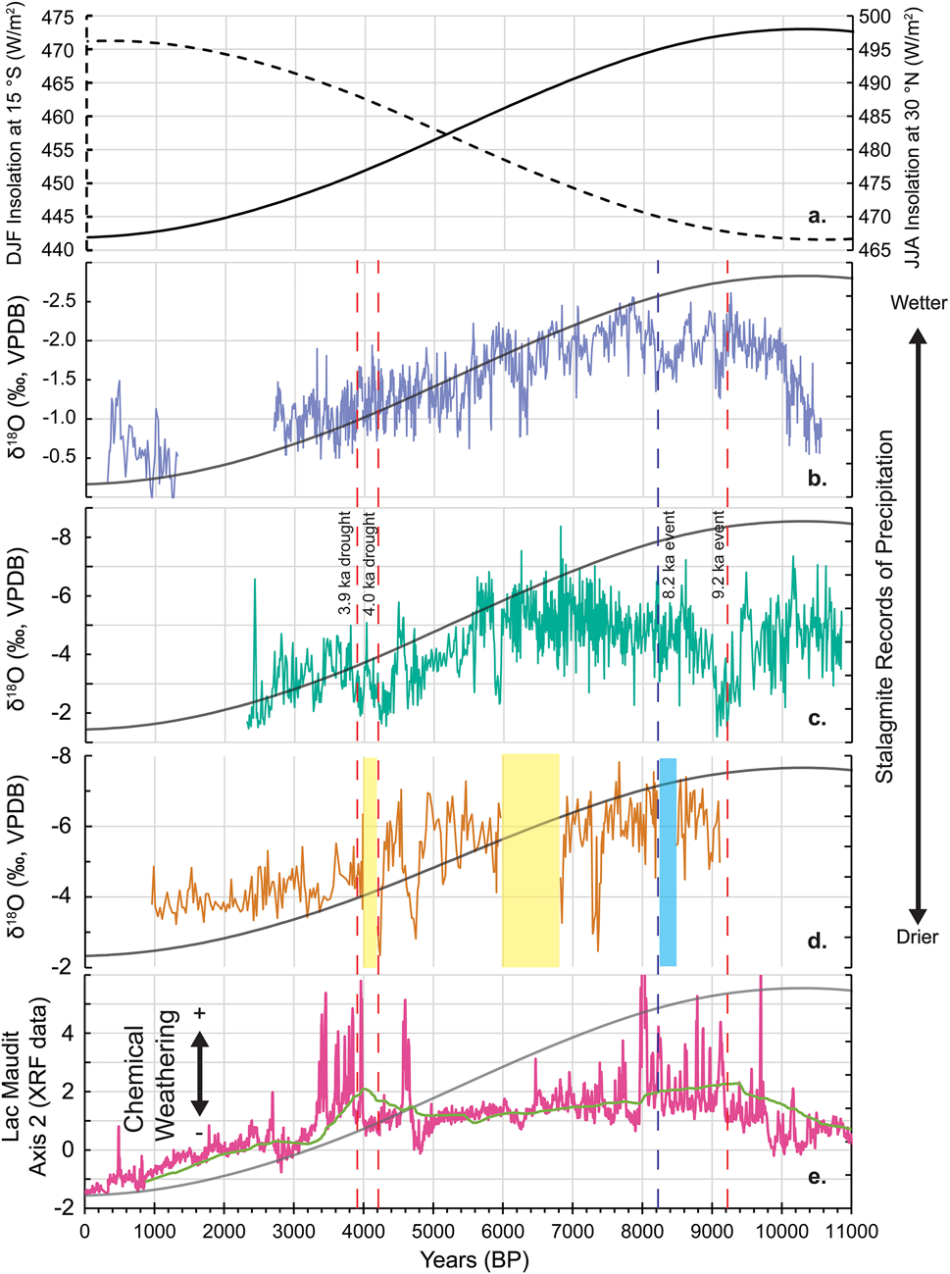
New and existing paleoclimate records from Northwestern Madagascar and Oman. (**a**) Incoming summer (DJF) solar insolation at 15°S latitude (Anjohibe, dashed line and axis) and summer (June–August, JJA) solar insolation at 30°N latitude (solid line and axis)^88^. (**b**) δ^18^O record from Qunf Cave, Oman at 17°N from Fleitmann et al.^36,58^. (**c**) AB11 δ^18^O record from Anjohibe (this study). (**d**) Anjohibe sample ANJ94-5 δ^18^O record from Wang et al.^30^ with dry hiatuses (yellow) and wet hiatus (light blue). (**e**) PCA Axis 2 based on XRF data from Lac Maudit core record from Teixeira et al.^31^. Lime green curve is a smoothed 200 year running average. For (**b**–**e**), 30°N insolation curve is also shown on right axes (same as in (**a**)) and red (dry) and blue (wet) dashed vertical lines denote events referenced in the main text.

Figure 4 shows Northern Hemisphere and Southern Hemisphere summer insolation curves (Fig. 4a), a Northern Hemisphere counterpart to Anjohibe (Fig. 4b), the AB11 record (Fig. 4c), another speleothem record from Anjohibe (Fig. 4d) and a recent sediment core X-ray fluorescence (XRF) record indicative of chemical weathering from Lac Maudit (12.6°S) in northern Madagascar (Fig. 4e)^30,31^. All three records of paleohydrology in the region track the Northern Hemisphere summer (JJA at 30°N) insolation curve and are antiphase to local insolation at the latitude of Anjohibe (~ 15°S) (Fig. 4a). These records indicate a response of tropical rainfall in the Southern Hemisphere to Northern Hemisphere forcing, which has also been observed in mainland Southeast Africa^57^. The observed time series of precipitation from Anjohibe is very similar to the pattern observed at Qunf Cave (Fig. 4b), located at 17°N in southern Oman^36,58^. Both records appear to broadly track northern hemisphere insolation with a more abrupt transition at ~ 6 ky BP to overall drier conditions. All Madagascar records show a wetter early to middle Holocene and drier late Holocene similar to the Northern Hemisphere trend observed in southern Oman. The timing of this middle Holocene transition to drier conditions in Madagascar begins ~ 6–4 ky BP and is likely different among records due to age model, location (alpine vs. lowland and location in the cave for speleothem records) as well as proxy differences. Regardless, the first-order parallel nature of these records indicates that rainfall in the Northern Hemisphere and Southern Hemisphere sectors of the western Indian Ocean are in phase with one another. The tropical rainfall belt in this section of the tropics must, therefore, be expanding and contracting^21^ or intensifying and weakening rather than shifting north and south with the insolation maximum.

The AB11 record also contains large (> 2‰) isotopic shifts at millennial and centennial scales. Centered at ~ 9.2 ky BP is a ~ 500 y long dry period in an otherwise wetter early Holocene compared to the late Holocene. At 9.442 ± 0.049 ky BP the δ^18^O values increase from − 5.71‰ to a maximum of − 1.19‰ at 9.060 ± 0.161 ky BP before returning to lower values of − 4.69‰ at 8.991 ± 0.193 ky BP. Our data provide additional evidence for a 9.2 ka event and extend the observed area of its occurrence into the Southern Hemisphere^59^. Notably, this 9.2 ka event is dry at Anjohibe (in phase with the Northern Hemisphere tropics) whereas the better known 8.2 ka event (discussed below), is wet at Anjohibe and antiphased with Northern Hemisphere records, though both are thought to be triggered by a North Atlantic melt-water pulse^39,60–62^. Therefore, teleconnections between high latitude forcings and Madagascar’s climate must differ for these two events. The 9.2 ka event in AB11 is considerably longer, and the amplitude of δ^18^O is greater than elsewhere^59^. It is not clear why the 9.2 ka event is so prominent in northwestern Madagascar. Possibly the 9.2 ka dry event is enhanced either by local forcings due to cooler SSTs in the western Indian Ocean or due to a negative IOD-like, enhanced SST gradient. More high-resolution SST records from both sides of the Indian Ocean basin that cover the early Holocene would be needed to test these hypotheses. The presence of aragonite mixed with calcite in some layers ~ 9.4 ky BP and older (1732 m and below) also suggests that this older period did include short dry periods. Previous authors who have worked in Anjohibe document aragonite as indicative of dry conditions due to its correlation with other proxies for dry conditions such as reduced layer-specific widths, type L (“Lessened”) surfaces, decreased growth rate and low luminescence and reflectance^30^. However none of the layers are pure aragonite in this older part of the record, while the younger portion (< 5 ky BP) does contain some layers of pure aragonite (Fig. 2, Supplementary Fig. S2) based on XRD. The mineralogy and isotopic evidence from AB11 show that despite short dry periods, and a dry 9.2 ka event, the early Holocene was still wetter than the late Holocene. Unfortunately, the one other longer speleothem record from Anjohibe^30^ does not contain material older than 9.1 ky BP (Fig. 4d) preventing comparison to our record, although the absence of growth might be evidence of relatively dry conditions.

Other centennial to millennial scale events detectable in our record include a wet period centered at approximately 8.2 ky BP. The 8.2 ka event is more globally distributed than the 9.2 ka event discussed above and is generally thought to result from a large melt-water pulse into the North Atlantic affecting thermohaline circulation and causing several hundred years of cold and dry conditions in the Northern Hemisphere tropics^60^. Tropical speleothem δ^18^O records show an antiphase relationship between precipitation changes in the Northern Hemisphere and Southern Hemisphere tropics during the 8.2 ka event, with model simulations suggesting this is due to a southward shift in the ITCZ^30^. More data are needed to understand how this event affected the Southern Hemisphere tropics, but previous studies by Voarintsoa et al.^61^ and Duan et al.^39^ (Supplementary Fig. S3) report two wet events in northwest Madagascar ~ 8.3 and 8.2 ky BP with 20 years of drier conditions in between. Our speleothem also records these brief ~ 100-year wetter periods (Supplementary Fig. S3) and supports the idea that, at least for the 8.2 ka event, the Southern Hemisphere tropics had an antiphase relationship with the Northern Hemisphere monsoonal regions^61,62^. The other longer Holocene record from Wang et al.^30^ has a hiatus (8.48–8.22 ky BP) during the 8.2 ka event, which the authors argue is actually wetter than any later parts of their record based on a type E (“Erosional”) surface, fast growth rates, and high reflectance and luminescence. Although these three records argue for a relatively wet 8.2 ka event, this ~ 100-year period does not stand out as anomalously wet when compared to the amplitude of centennial variability in the rest of the AB11 Holocene record (Fig. 4).

In fact, the wettest (most depleted δ^18^O) part of our record is middle Holocene (~ 6.8 ky BP) and appears to be part of the centennial to decadal variability seen throughout this older and wetter part of the record (~ 6.2 to 7.6 ky BP). Of note is the fact that the other longer Holocene record from Wang et al.^30^ (Fig. 4d) suggests that it’s actually quite dry during this time period with higher δ^18^O (~ − 4.5‰) and then a hiatus (6.81–5.98 ky BP) they argue is due to dry conditions based on a basal type L (“Lessened”) surface, slower growth rates, and low reflectance and luminescence. However the top surface of this hiatus is a type E (“Erosional”) surface, attributed to wet conditions. The differences seen between these two longer Holocene records (Fig. 4c,d) stress the importance of comparing multiple stalagmites from the same cave. For example the hiatus seen in one record (Fig. 4d) might reflect changes in flow path in the epikarst as opposed to larger climate driven changes to regional precipitation. The differences in AB11 and the record from Wang et al.^30^ could also be related to local cave effects such as rates of degassing, evaporation or other kinetic effects due to their different locations of growth in Anjohibe. While we can’t say for certain the cause of centennial to millennial scale discrepancies between AB11 and ANJ94-5 from Wang et al.^30^, the longer-term trend (wet early to middle Holocene, dry late Holocene) and the corresponding range in δ^18^O values (~ − 8 to − 1‰) for AB11 and (~ − 8 to − 2‰) for ANJ94-5 are comparable (Fig. 4).

Another time period that has been studied extensively in other speleothem records is the 4.2 ka event, which despite debatable global expression, unknown forcing mechanisms, and temporal variability among different records, is often described as two dry events ~ 4.2 ky BP and 3.9 ky BP with a wet or return to ‘normal’ conditions between 4.1 and 4.0 ky BP^63,64^. The previously published Wang et al.^30^ record contains a dry hiatus during this time period as does a record from nearby Anjohikely^24^. Our AB11 δ^18^O record does suggest two drier time periods ~ 4.3 ky BP and 3.9 ky BP, but they do not stand out as particularly abrupt or severe (Fig. 4c). The first dry event begins at 4.495 ± 0.049 ky BP with δ^18^O values of − 5.78‰, and maximum drying indicated by δ^18^O values of − 1.56‰ at 4.312 ± 0.078 ky BP. This first dry event ends at 4.033 ± 0.119 ky BP with lower δ^18^O values of − 5.08‰, but then a return to drier conditions is marked by higher δ^18^O values of − 1.91‰ at 3.940 ± 0.124 ky BP. This second dry event ends at 3.802 ± 0.121 ky BP, with a return to lower δ^18^O values of − 4.99‰. Another confirmation of the climate signal in our AB11 δ^18^O record is the agreement between AB11 and another higher resolution record from Anjohibe, stalagmite AB13^65^, which spans this “4.2 ka event” (Supplementary Fig. S2). Interestingly, dry conditions in both the Southern and Northern Hemisphere tropics^63^ suggest that the mechanism for the drying is not simply the migration of the ITCZ, and variability in the expression and timing of these events in speleothem records of the Indian Ocean Basin suggest the “4.2 ka event” is more nuanced than the 8.2 ka event^24,64^.

### Why does rainfall in NW Madagascar follow NH summer insolation?

**Figure 5:**
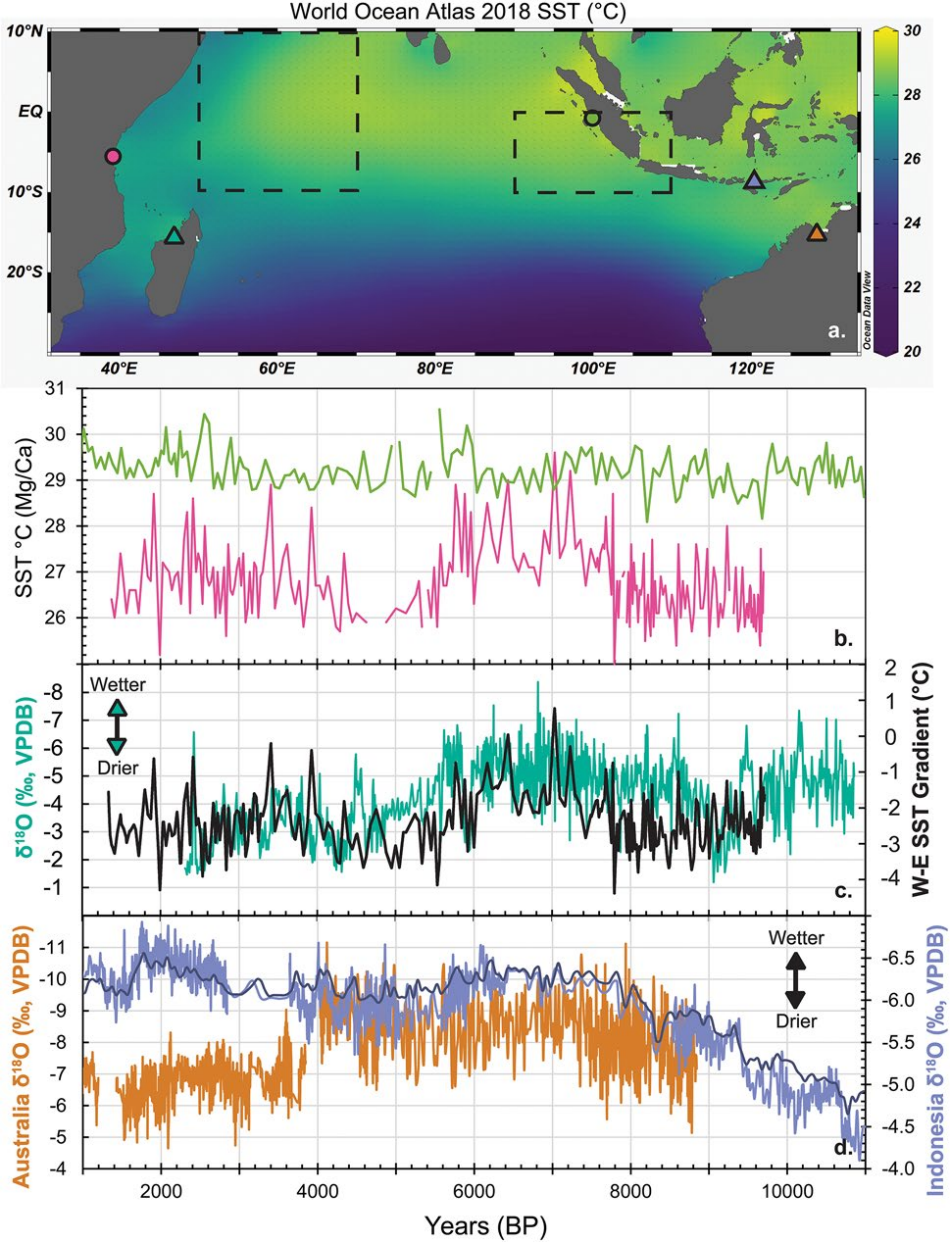
(**a**) World Ocean Atlas 2018 (WOA18) SSTs for decades between 1955–2017^89,90^. Data visualization via Ocean Data View version 4, downloaded from https://odv.awi.de^91^. Areas used to calculate DMI outlined in dashed lines. Sites of ocean gravity and piston cores shown as circles with colors matching Mg/Ca SST records in (**b**)^70,71^. (**c**) AB11 δ^18^O record from Anjohibe (this study, blue green) and W to E SST gradient (black) for the Indian Ocean based on records in (**b**). (**d**) Speleothem δ^18^O records from Western Australia (KNI-51)^75^ and Flores, Indonesia (Liang Luar). Light purple is record from Griffiths et al.^49^ and dark purple is 50-year sea-water corrected composite record from Scroxton et al.^52^.

As noted, the first order climate trend documented in stalagmite AB11 and also present in stalagmite ANJ94-5^30^ and the Lake Maudit sedimentary record^31^ is decreasing available moisture across the Holocene that roughly parallels Northern Hemisphere summer insolation. This trend is in phase with Northern Hemisphere Holocene monsoon records^36,53,54,58^. Holocene paleoclimate in northwestern Madagascar cannot, therefore, be explained by southward, meridional migration of the ITCZ and tropical rain belt as expected in the Global Paleomonsoon paradigm. Studies in Madagascar at multi-decadal scale in the late Holocene^6^ and during Heinrich Event 1^66^ have also noted in phase climate variations between Madagascar and Northern Hemisphere records and suggested that east to west SST gradients in the Indian Ocean, with warming in the west relative to the east, are associated with greater rainfall in the study area. In addition, studies in East Africa^13,67,68^ have found that, on orbital to centennial timescales, western Indian Ocean SSTs play a dominant role in enhancing moisture transport and rainfall over the region, similar to what is observed during the annual cycle today^69^. Therefore, we also investigate a possible link to Indian Ocean SSTs and zonal SST gradients to see if they have any relationship to the AB11 δ^18^O record. High resolution SST records over the Holocene are not numerous but two foraminiferal Mg/Ca based SST records from off the coast of Tanzania (GeoB 12605)^70^ and western Sumatra (SO189-39KL)^71^ allow us to compare SSTs and SST gradients across the Indian Ocean (Fig. 5a) over most of the time period AB11 grew (Fig. 5b). Generally, eastern Indian Ocean SSTs are relatively constant at ~ 29 °C, but the western Indian Ocean SSTs fluctuate with a cooler early Holocene (~ 26 °C), warmer middle Holocene (7.8–5.8 ky BP) and then cooler SSTs for the rest of the record (Fig. 5b).

Comparing the AB11 δ^18^O record to the SST records shows that when western Indian Ocean SSTs are higher, rainfall increases (more negative δ^18^O) in northwestern Madagascar and vice versa (Fig. 5b,c). This relationship could be due to basic thermodynamic controls whereby warmer local SSTs leads to increased tropical precipitation^72^. However there is also a relationship between AB11 δ^18^O and the W to E SST gradient using the two Mg/Ca records (Fig. 5c). These core sites lie within or near the areas (Fig. 5a) used to calculate the SST anomalies (e.g. Dipole Model Index), used for characterizing the modern IOD and associated Walker Circulation. This relationship could be due to a processes similar to modern positive IOD events, with enhance easterlies, bringing warm SST anomalies to the tropical western Indian Ocean, reducing the W to E SST gradient (warming in the west relative to the east) and wetter conditions in East Africa^8,10^. To determine whether western Indian Ocean SSTs or the W to E SST gradient control Madagascar precipitation we look at the Spearman correlation coefficient between these records (Fig. 5b,c) and AB11 δ^18^O. The relationship between AB11 δ^18^O and the W to E SST gradient suggests a significant relationship (Fig. 5c, ρ = − 0.30, p ≪ 0.01), which is stronger than the correlation with just the western Indian Ocean SSTs (Fig. 5a, ρ = − 0.26, p ≪ 0.01). Therefore, it appears the gradient plays a greater role in modulating Anjohibe precipitation.

The good correlation between the W to E SST gradient and precipitation in northwest Madagascar suggests a strong zonal rather than meridional control on rainfall. On millennial to orbital timescales, the tropical rainfall belt in the western Indian Ocean is not impacted by meridional migration of the mean ITCZ location, but by a zonal Walker circulation mechanism. From ~ 8 to 6 ky BP, the W to E SST gradient is reduced, driven by warming in the western Indian Ocean rather than upwelling and cool SST anomalies in the east (Fig. 5b,c). This is unlike modern inter-annual positive IOD events, which are initiated by upwelling in the east^8^, suggesting a different mechanism for the SST gradient change on orbital to millennial timescales. Unlike today, with a more negative IOD mean state, this could reflect a more positive IOD mean state during the middle Holocene as suggested by other records (summarized in Abram et al.)^10^, though the timing in these other records is slightly younger (5.6–4.2 ky BP). From ~ 6 to 5 ky BP, the SST gradient becomes stronger, again driven by cooling in the western Indian Ocean (Fig. 5b,c). Why this transition occurred is not clear, but it does take place (~ 5 ky BP) close to when Southern Hemisphere summer insolation begins to exceed Northern Hemisphere summer insolation (Fig. 4a). Whether this association is coincidental or reflects a causal relationship remains to be determined. Climate models have found precession minima (perihelion during Northern Hemisphere summer) create positive IOD-like conditions (e.g. stronger easterlies, warmer SSTs and increased precipitation) in the western Indian Ocean^73,74^. Thus, it appears that while Northern Hemisphere insolation likely contributes to the pattern of precipitation (wet early Holocene, dry late Holocene) that we observe in Southern Hemisphere sites (Fig. 4), the zonal SST gradient in the Indian Ocean plays a more dominant role in modulating precipitation in northwestern Madagascar. When the zonal gradient is weak (near zero), we see wet conditions at Anjohibe and when the gradient is strong (away from zero) we have drier conditions at Anjohibe (Fig. 5c). For the middle to late Holocene, the effects of decreasing Northern Hemisphere insolation and a strengthening of the zonal gradient work in the same direction, leading to drier conditions. However in the early Holocene (> 8 ka) a strong gradient would lead to drier conditions at Anjohibe while higher Northern Hemisphere summer insolation should result in wetter conditions. This is likely why, despite the same long-term similarities between the Oman record and the ones from Anjohibe, there are millennial to centennial scale differences at these two locations in the early Holocene as these two precipitation drivers superimpose one another at Anjohibe.

While involving a zonal SST gradient similar to the modern-day IOD, these observations show that the IOD mechanism operating today cannot explain the much longer timescale patterns over the Holocene. For example, we do not observe the typical opposite pattern of precipitation anomalies between Indonesia and East Africa^8^. Indonesia and Western Australia are not dry during the middle Holocene (~ 8–6 ky BP) when there is a weaker W to E SST gradient and northwestern Madagascar is wetter. Instead, speleothem records^49,52,75^ show relatively wet conditions during that time (Fig. 5d) on both sides of the Indian Ocean. Also, these regions on the eastern edge of the Indian Ocean are not uniformly wetter in the late Holocene when the W to E SST gradient is stronger (Fig. 5d). Indonesia appears wet at this time while Australia is drier compared to earlier in the Holocene (Fig. 5d).

## Conclusion

The AB11 speleothem record supports other long Holocene records of paleoclimate in northwestern Madagascar including other speleothem records from Anjohibe^30^ and a higher elevation lake record^31^. These records all show a wetter early Holocene transitioning to the drier conditions of today, with AB11 and Qunf Cave in Oman showing a more abrupt shift at ~ 6 ky BP (Fig. 4). On multi-millennial timescales northwestern Madagascar is in phase with the Northern Hemisphere tropics. However, during the 8.2 ka event, precipitation in Madagascar is antiphase to the Northern Hemisphere, suggesting the migration of the ITCZ is the main climate driver during this short period. However, during other centennial to multi-centennial events like the 9.2 ka and “4.2 ka” event, similarly dry conditions in both northwestern Madagascar and the Northern Hemisphere tropics are inconsistent with a southward shift of the ITCZ. Therefore, we argue that meridional shifts of the ITCZ are not the dominant control on this region’s precipitation. Instead, we argue that zonal gradients in Indian Ocean SSTs (Fig. 5c) play a more important role in triggering changes in Holocene precipitation in northwestern Madagascar. Projections of future warming due to anthropogenic climate change in the Indian Ocean suggest stronger easterlies, greater warming in the western Indian Ocean and a shoaling of the equatorial thermocline^10,11^. Compared to pre-industrial conditions, this could indicate the region is shifting to a more positive-IOD mean state not unlike conditions we observe in the middle Holocene (~ 8–6 ky BP).

## Methods

### Stable isotope analysis

After AB11 was cut and polished, we used a micro-mill to drill powders for stable isotope analysis at 1 mm increments between 0 and 388 mm. Lower than this depth, the diameter of the stalagmite increased and was sampled at 2 mm increments. A total of 1184 powdered samples was collected for stable isotope analyses. Measurement of AB11 δ^13^C and δ^18^O was performed at the Stable Isotope Laboratory at the University of Massachusetts on a Thermo Scientific Delta V Isotope Ratio Mass Spectrometer (IRMS) with an on-line carbonate preparation system, (Gasbench II). Results are reported as permil (‰) relative to the Vienna PeeDee Belemnite (VPDB) standard. Reproducibility of in-house standard materials for individual sample runs is 0.07‰ for δ^13^C (n = 7) and 0.09‰ for δ^18^O (n = 8) or better. Wavelet spectral analysis was performed using the R package biwavelet^76–78^ and the isotope record was interpolated using the Akima splines method^79^ with the R package Pracma^80^. For comparison of our AB11 δ^18^O record to SSTs, we linearly interpolated both the Tanzania and Sumatra Mg/Ca SST records and our AB11 δ^18^O record to annual resolution. We note that the error on the SST records can be quite large, with ± 1σ error up to 150 years, making any real relationship to a decadal or sub-decadal mode like the modern IOD impossible.

### X-ray diffraction analysis

Additional powders were drilled adjacent to the isotope analysis pits for XRD analysis at Smith College using a Rigaku SmartLab SE Diffraction System. The percentage of calcite and aragonite was approximated based on peak intensity for the 111 aragonite peak and 104 calcite peak compared to the total intensity for both peaks.

### U/Th geochronology

All geochronology work was done at the Massachusetts Institute of Technology clean lab. Thirty-one samples weighing ~ 200 mg were drilled from AB11 with a vertical mill. Once the powders were dissolved and spiked with a ^229^Th–^233^U–^236^U tracer, U and Th were isolated by Fe co-precipitation and purified with ion-exchange chromatography using columns containing AG1-X8 resin following^81^. The subsequent fractions were analyzed alongside a total procedural blank using a Nu Plasma II-ES MC-ICP-MS at MIT with the methods detailed in a prior study on a speleothem from Anjohibe^29^. An initial ^230^Th/^232^Th ratio of 4.4 ± 2.2 × 10^−6^ was used based on the average upper continental crust composition. The ^230^Th and ^234^U half-lives from^82^ and the ^238^U half-life from^83^ were used in age calculations. COPRA^84^ was used to build an age model and develop proxy-age relationships using 1000 Monte Carlo simulations. Six samples were removed from the Monte Carlo simulations due to large 2σ errors of 1000 years or more or due to age inversions.

### Supplementary Information

Supplementary Information 1.

Supplementary Information 2.

## Data Availability

The datasets generated and analyzed during the current study are available in the National Oceanic and Atmospheric Administration (NOAA) Paleoclimatology Data Archive available online at https://www.ncei.noaa.gov/access/paleo-search/study/38600.
