# Supplementary material for: Zonal control on Holocene precipitation in northwestern Madagascar based on a stalagmite from Anjohibe
Source: Sci Rep. 2024 Mar 6;14:5496. doi: 10.1038/s41598-024-55909-6 (PMC10917758; doi:10.1038/s41598-024-55909-6)
Supplement: Supplementary file 1 — Supplementary Information 1. [file 41598_2024_55909_MOESM1_ESM.docx]

**Zonal control on Holocene precipitation in northwestern Madagascar based on a stalagmite from Anjohibe**

**Supplementary Information**

**Robin R. Dawson**^1,*^**, Stephen J. Burns**^1^**, Benjamin H. Tiger**^2,3^**, David McGee**^2^**, Peterson Faina**^4^**, Nick Scroxton**^5^**, Laurie R. Godfrey**^6^ **and Lovasoa Ranivoharimanana**^7^

1 University of Massachusetts Amherst, Department of Earth, Geographic and Climate Sciences, Amherst, MA 01003, USA

2 Massachusetts Institute of Technology, Department of Earth, Atmospheric, and Planetary Sciences, Cambridge, MA, 02139, USA.

3 Woods Hole Oceanographic Institution, Department of Geology and Geophysics, Woods Hole, MA, 02543, USA.

4 The Climate School, Columbia University, New York, NY 10025, USA

5 Maynooth University, Irish Climate Analysis and Research Units, Department of Geography, Maynooth, Ireland

6 University of Massachusetts, Department of Anthropology, Amherst, MA 01003, USA

^7^ Université D’Antananarivo Mention Bassins Sédimentaires, Evolution, Conservation, Faculté des Sciences, Antananarivo, Madagascar

*Corresponding author: Robin R. Dawson (rrdawson@umass.edu)

**Supplementary Figure S1:** Wavelet spectral power analysis of AB11 δ^18^O record

**Supplementary Figure S2:** δ^18^O and δ^13^C values of AB11 calculated assuming 100% calcite and 100% aragonite and estimates of mineralogy based on XRD.

**Supplementary Figure S3:** Replicate stalagmite δ^18^O records from Anjohibe.

**Supplementary Data File (separate Excel spreadsheet):** Chronology Tab: U/Th Data; Data Tab: Stable Isotope (δ^13^C, δ^18^O) and XRD (% aragonite) Data. Available in the National Oceanic and Atmospheric Administration (NOAA) Paleoclimatology Data Archive available online ([https://www.ncei.noaa.gov/access/paleo-search/study/38600](https://nam10.safelinks.protection.outlook.com/?url=https%3A%2F%2Fwww.ncei.noaa.gov%2Faccess%2Fpaleo-search%2Fstudy%2F38600&data=05%7C01%7Crrdawson%40umass.edu%7Cba010544d54842b3050d08dbc4f247dc%7C7bd08b0b33954dc194bbd0b2e56a497f%7C0%7C0%7C638320317108210484%7CUnknown%7CTWFpbGZsb3d8eyJWIjoiMC4wLjAwMDAiLCJQIjoiV2luMzIiLCJBTiI6Ik1haWwiLCJXVCI6Mn0%3D%7C3000%7C%7C%7C&sdata=pci2KkRZ8TTXpGnas3KsGmJz6g1P%2Foqj1%2FZhhCaLj8A%3D&reserved=0)).

**
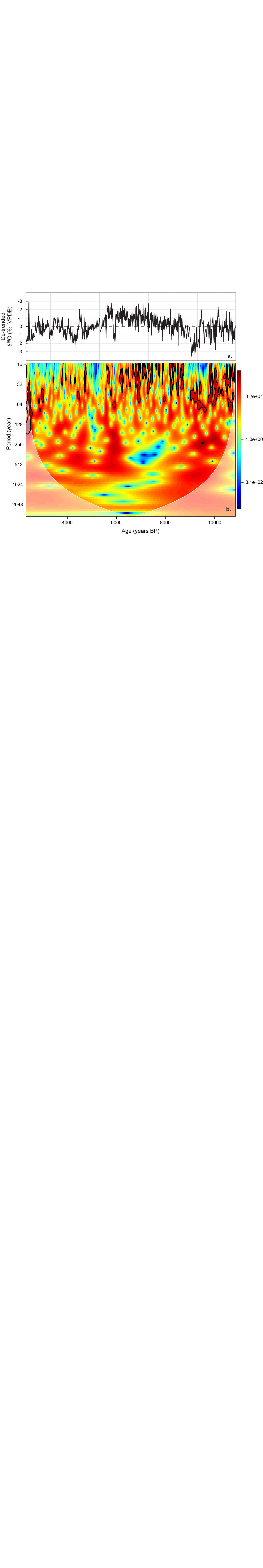
Supplementary Figure S1.** Wavelet spectral power analysis of AB11 δ^18^O record. (a) Interpolated and de-trended AB11 δ^18^O time series. (b) Wavelet power spectrum of AB11 δ^18^O record with warmer colors indicating higher power with black lines encircling frequencies that exceed the 90% confidence level.


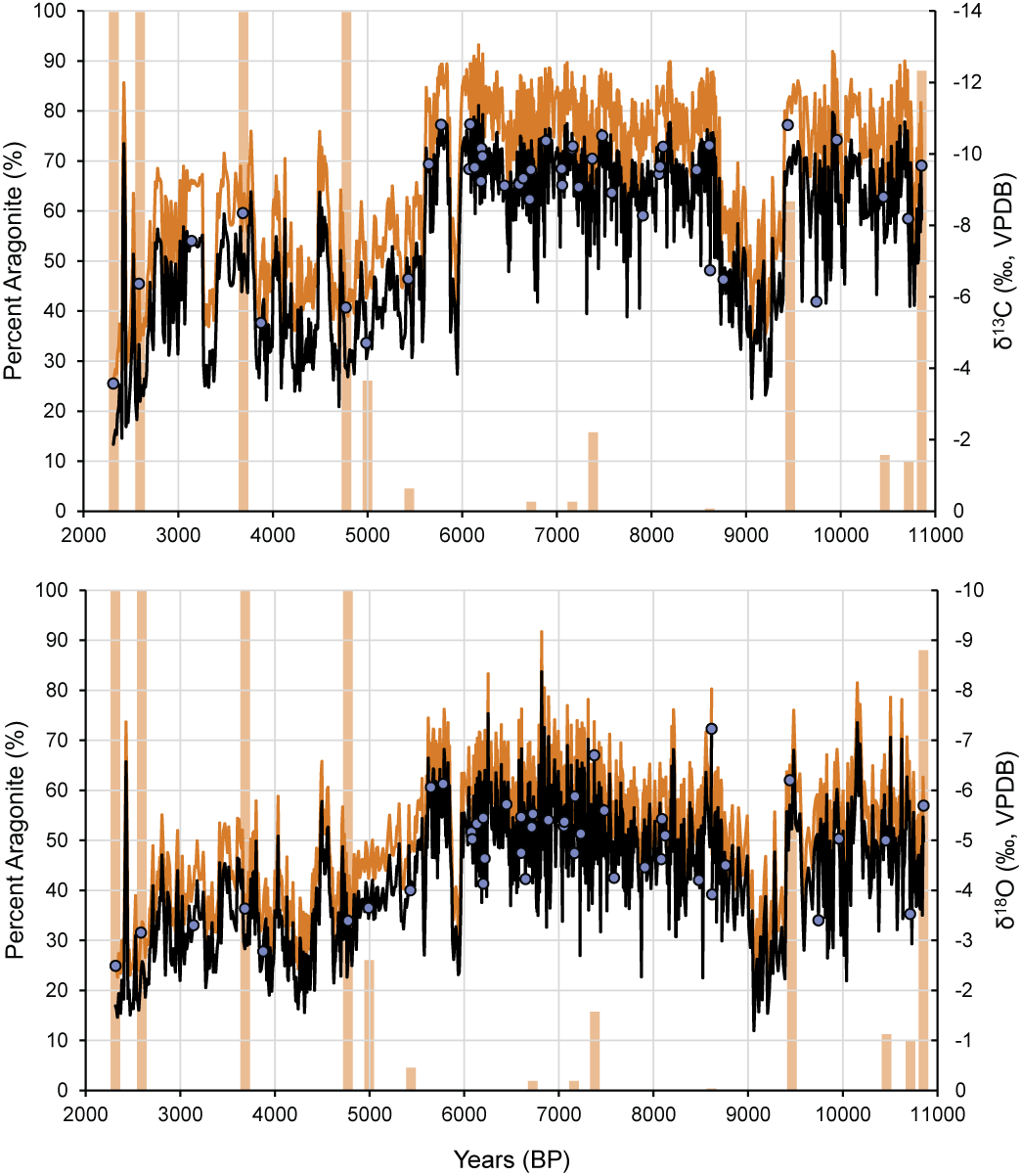


**Supplementary Figure S2.** Stable carbon (top) and oxygen (bottom) isotope values of AB11 calculated assuming a calcite composition (black) and aragonite corrected values (orange) using fractionation factors from Romanek et al., 1992; Kim and O’Neil, 1997; and Kim et al., 2007. Selected depths sampled for mineralogical analysis using XRD show the estimated % aragonite in orange bars. Purple circles are the corrected δ^13^C and δ^18^O values assuming the corresponding proportion of calcite and aragonite determined by XRD. When a purple circle has no corresponding bar beneath it, the composition from XRD was 100% calcite. Some XRD sample depths lie between those for isotopic analysis, so mineralogy was assumed to be the same 1 mm above and below the XRD sample depth. When 100% calcite is present the purple circles overlap the black curve and when 100% aragonite is present they overlap the orange curve. When the composition is mixed the isotope values are proportionally intermediate.

**
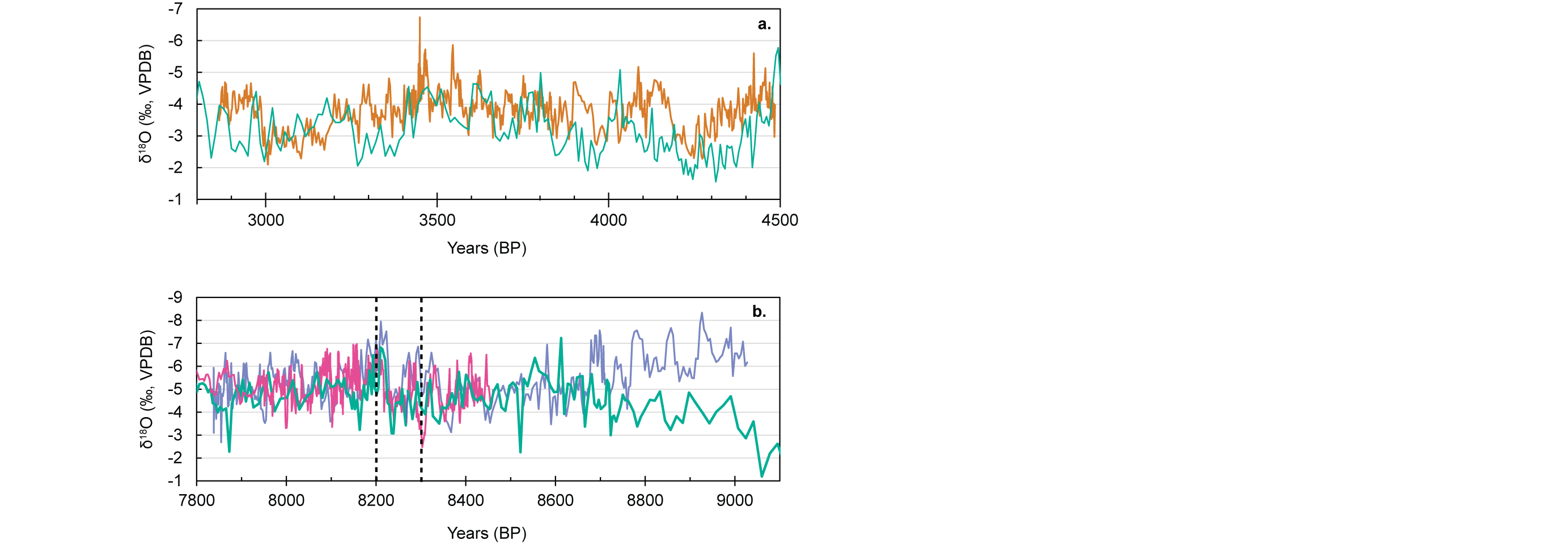
Supplementary Figure S3.** Replicate stalagmite δ^18^O records from Anjohibe. (a) Anjohibe δ^18^O record from stalagmite AB13 (orange) from Williams et al. (2024) and AB11, this study (blue green). (b) Anjohibe δ^18^O records from stalagmites ANJB-2 (purple) from Voarintsoa et al., 2019, ABC-1 (pink) from Duan et al., 2021, and AB11, this study (blue green) with the two subevents at 8.2 ka and 8.3 ka indicated with vertical dashed lines.
